# Editorial: Sentinel CLECs at Immunological Decision Nodes

**DOI:** 10.3389/fimmu.2020.02066

**Published:** 2020-09-10

**Authors:** J. Kenneth Hoober, Sandra J. van Vliet, Diana Dudziak, Laura L. Eggink

**Affiliations:** ^1^Susavion Biosciences, Inc., Tempe, AZ, United States; ^2^Amsterdam University Medical Center, Vrije Universiteit Amsterdam, Amsterdam, Netherlands; ^3^Department of Molecular Cell Biology and Immunology, Cancer Center Amsterdam, Amsterdam Infection & Immunity Institute, Amsterdam, Netherlands; ^4^Laboratory of Dendritic Cell Biology, Department of Dermatology, Friedrich-Alexander University of Erlangen-Nürnberg (FAU), University Hospital Erlangen, Erlangen, Germany; ^5^Deutsches Zentrum Immuntherapie (DZI), Friedrich-Alexander University of Erlangen-Nürnberg (FAU), University Hospital Erlangen, Erlangen, Germany; ^6^Comprehensive Cancer Center Erlangen-European Metropolitan Area of Nuremberg (CCCER-EMN), Friedrich-Alexander University of Erlangen-Nürnberg (FAU), University Hospital Erlangen, Erlangen, Germany; ^7^Medical Immunology Campus Erlangen, Friedrich-Alexander University of Erlangen-Nürnberg (FAU), University Hospital Erlangen, Erlangen, Germany

**Keywords:** sentinel receptors, CLEC receptors, immunomodulation, dendritic cells, ligand diversity

It is traditional in immunology to characterize cell type polarization events by specifying either (i) the perceived causative transcription factors or (ii) the environmental cytokine milieu that is necessary to modulate cell type phenotypic transitions. Such factors are considered to give rise to cell surface receptor populations that then, somewhat uniquely, define myeloid and lymphoid functionality.

The evolving ability to maintain functional integrity when under constant threat is a defining feature of living organisms and their immune systems. Success is dependent on the ability to both recognize and respond to a constellation of environmental stressors. While such stressors can be either non-self or self in origin, their cellular perception sets in motion immune responses that, by polarizing various cell subpopulations, seek to mitigate, or eliminate perceived threats.

C-type (Ca^2+^-dependent) lectin (CLEC) receptors are integral to many critical aspects of the immune system. The spectrum of immunological responses that environmental stressors can evoke include (i) Ca^2+^ mobilization; (ii) ectodomain receptor shedding, and (iii) immunogenetic reprogramming. Since most endocytic CLEC receptors require Ca^2+^ for recognition of the ligand, their release from the receptor after internalization into an early endosome constitutively initiates a Ca^2+^ signal. Owing to their structural flexibility and adaptive potential, members of the CLEC superfamily are uniquely positioned at critical immunological checkpoints on myeloid and lymphoid cell types. These receptors influence the balance between appropriate immunogenic or immuno-tolerant responses.

CLECs are the largest and most diverse lectin-type receptor family. The diversity is well-reflected in the two research articles and six reviews in this Research Topic. Although these articles describe members of the C-type lectin family, which are tied together by phylogeny and structural similarities, several receptors that are included do not bind sugars (thus, not technically lectins) nor do all require Ca^2+^ to bind their ligand.

Two research papers in this collection describe responses of monocyte-derived dendritic cells to novel ligands for MGL, also called CLEC10A. Zaal et al. synthesized glycodendrimers carrying the MGL ligands N-acetylgalactosamine or N-acetylgalactosamine(β1-4)galactose. The surprising results from these experiments show that these ligands evoke differential reprogramming of dendritic cells, even though they bind the same MGL receptor. Moreover, triggering of the MGL receptor lowered the glycolytic activity in dendritic cells, suggestive of a more quiescent metabolic and tolerogenic phenotype. Stolk et al. prepared liposomes that incorporated α-galactosylceramide or palmitoylated Le^Y^ as ligands to target C-type lectin receptors on dendritic cells (DC-SIGN) and Langerhans cells (langerin) and that were loaded with palmitoylated peptide antigens. This construct was taken up by dendritic cells, which cross-presented the peptide antigen to T cells to generate tumor specific CD8^+^ T and iNKT cells. This approach is offered as a novel strategy for vaccination to induce high level immunity.

The other six articles in the Research Topic review basic aspects of C-type lectin receptors. Cueto et al. describe the current knowledge of the important receptor, DNGR-1. As a marker for type 1 conventional dendritic cells (cDC1), CLEC9A, commonly referred to as DNGR-1, recognizes F-actin that is exposed during cell necrosis. Antigens are effectively presented to CD8^+^ T cells, but DNGR-1 does not classically activate DCs. Instead, DNGR-1 has an anti-inflammatory role by activating the phosphatase SHP-1, which limits signaling and the recruitment of neutrophils to injured tissues. This review highlights the versatility of DNGR-1 in promoting cross-presentation or restraining inflammation in different environments.

Pires et al. provide a comprehensive review of remarkable studies on reprogramming cells for regenerative medicine. Extensive work is described that identified transcription factors that impose a DC fate in fibroblasts. Evidence of success in reprogramming fibroblasts was the expression of CLEC9A, a specific marker for cDC1 DCs. The ability to reprogram immune cells should provide new tools for cancer immunotherapy and manipulation of the immune system in health and disease. Drouin et al. review the structure and function of C-type lectin-like receptors of the “Dectin family.” CLEC4E (MINCLE), CLEC8A (LOX-1), CLEC9A (DNGR-1), and CLEC12A (MICL) are indeed “sentinels.” These four do not bind sugars but rather damage-associated molecular patterns (DAMPs) that arise from necrotic or dead cells. CLEC4E binds glycolipids, CLEC8A binds oxidized low-density lipoproteins, CLEC9A binds F-actin and CLEC12A binds crystals of sodium urate. This discussion provides an excellent view into the variety of receptors that nevertheless structurally contain similar binding domains. When the receptor does bind Ca^2+^, the cation plays a structural role rather than a direct interaction with the ligand. The review by Sung and Hsieh extends the discussion of “sentinel” C-type lectin-like receptors that recognize acute viral infections. CLEC2 and CLEC5A also do not bind sugar-based ligands but rather virus particles such as dengue virus and H5N1 influenza virus. This review also emphasizes the interaction of different CLEC receptors, how these receptors interact with toll-like receptors, and the activation responses that are mediated by Syk. This discussion is particularly relevant in regard to the current threat of major viral infections.

Whereas, the previously listed reviews describe “sentinel” C-type lectin-like receptors that bind cellular material other than glycans, the review by Busold et al. comprehensively discusses the role of glycans and C-type lectin receptors in shaping immune responses. A particular emphasis is the use of liposome adjuvants and glycoconjugates to deliver drugs to specific targets on DCs. The review by Hoober et al. discusses several C-type receptors that induce variable responses depending upon the nature of the ligand. Different signals result from the size of the ligand, which can lead to aggregation of the receptor. Size of the ligand can also determine the length of time required to process the digestive products for presentation on the cell surface as MHC class I and II complexes. Furthermore, the extent of engagement of a receptor by the ligand can determine activation or induction of tolerance of DCs.

C-Type lectins contain one or more immunoreceptor tyrosine-based activation motif (ITAM), immunoreceptor tyrosine-based inhibitory motif (ITIM), or a hemITAM, which also serves as an endocytic motif, in the cytoplasmic domain. When a motif is absent, the receptor functions with an adaptor protein, which often contains an ITAM. Collectively, the articles in this Research Topic cogently present the spectrum of characteristics of C-type lectin receptors on immune cells.

## Author Contributions

The draft of the Editorial was written by JH. It was read and edited by SV, DD, and LE. All authors contributed to the article and approved the submitted version.

## Conflict of Interest

JH and LE are co-founders of Susavion Biosciences, Inc. The remaining authors declare that the research was conducted in the absence of any commercial or financial relationships that could be construed as a potential conflict of interest.

